# Using Culturally Relevant Meal Kits to Improve Cooking Skills, Reduce Food Waste, and Promote Engagement with a Campus Food Access Resource: An Exploratory Pilot Study

**DOI:** 10.3390/nu17050843

**Published:** 2025-02-28

**Authors:** Isabella Remolina, Melissa J. Teuber, Ellie Lee, Deborah S. Fetter

**Affiliations:** 1 Department of Public Health, University of California, Davis, Davis, CA 95616, USA; iremolina@ucdavis.edu; 2 Department of Nutrition, University of California, Davis, Davis, CA 95616, USA; mjteuber@ucdavis.edu (M.J.T.); elelee@ucdavis.edu (E.L.)

**Keywords:** food insecurity, meal kits, college students

## Abstract

**Background/Objective:** Students’ taste preferences, cooking skills, and cultural backgrounds impact their use of food access resources on campus. Meal kits include pre-sorted ingredients, which could address food waste and help to prepare meals with unfamiliar ingredients. The objective of this exploratory pilot study was to develop and investigate the impact of culturally relevant meal kits on cooking skills, food waste, and food security tailored to UC Davis students. **Methods:** Meal kits included ingredients found at the campus food pantry. Three culturally relevant recipes were selected: High-Protein Avocado Toast, Mexican-Inspired Quinoa Bowl, and a Korean Vegetable Stir-Fry. Students were randomly assigned to the intervention meal kit group (n = 50), while the comparison recipe card group received a digital recipe card (n = 25). Data were collected through pre- and post-surveys administered online, in addition to open-ended, qualitative feedback through surveys after each meal kit or recipe card. **Results:** Thirty-two participants in the meal kit group and four participants in the recipe card group completed all study measures. Both groups experienced an increase in being classified as high food security over the three-week intervention period (+13% in the intervention group and +75% in the comparison group). Further, the intervention group improved cooking self-efficacy (+1.2 points; *p* < 0.01) and food waste practices. Participants appreciated the meal kits’ ease of preparation, clear instructions, and minimal cooking steps. **Conclusions:** The preliminary findings of this exploratory pilot study highlight the potential importance of culturally relevant interventions to address food security and promote healthier eating habits among college students. However, more research is needed with a larger, more diverse sample over a longer duration.

## 1. Introduction

Food insecurity remains a major issue, especially on college campuses where an estimated one in every four college students experience food insecurity [1]. Food security refers to having access to nutritious, culturally preferable foods, and being able to use them [2]. The lack of accessible nutrient-dense food can be detrimental to a college student’s physical and mental health, in addition to academic performance [1]. Health consequences associated with food insecurity include an increased risk of type II diabetes, hypertension, heart disease, and excess adiposity, among others [3]. College students face high food insecurity rates due to competing expenses, including high tuition costs and other financial expenses, limited food access, and lack of time to cook/prepare foods [4]. Despite most college students having some general knowledge of nutritional needs, many students tend to choose less nutrient-dense food options due to convenience and taste [5].

Due to busy schedules, college students report not having enough time to prepare nutritious meals at home. Thus, many students rely on easily accessible food options, such as microwavable foods, foods from dining halls, fast food, and/or restaurants [5]. Further, individuals who consume more fast food are less likely to cook at home [6]. The nutrition content of readymade meals, including fast food, is typically high in sodium, fat, and/or calories [5]. Previous research has shown that a high intake of prepared and highly processed foods is linked to poor nutrient intake and increased calorie consumption, contributing to negative health outcomes [7]. According to the 2020–2025 Dietary Guidelines for Americans, it is recommended to limit the intake of sodium, fat, and added sugars to help reduce the risk of a variety of diet-related chronic diseases, such as cardiovascular disease and certain cancers [8].

Many college campuses aim to improve food security through campus gardens, food assistance programs, cooking classes, and food pantries [9]. However, students’ taste preferences, cooking skills, and cultural backgrounds impact their use of food access resources on campus [9]. Research has shown that when these factors are taken into consideration, the effectiveness of food access programs can be significantly improved [10]. A recent study conducted at the University of California, Berkeley, evaluated a semester-long food skills course in partnership with a teaching kitchen [10]. From this study, participants experienced an increase in cooking self-efficacy, increased vegetable intake, and more frequent cooking. These campus interventions suggest that when students are given food resources and education to develop cooking skills, they are more likely to prepare and consume nutrient-dense meals [10].

An example of a campus initiative with the mission to improve food security is the Associated Students, University of California, Davis, (ASUCD) Pantry, referred to as “The Pantry”. This is a student-operated food access resource at UC Davis created in 2010 to address the growing challenges students face in accessing essential food resources while pursuing their academic endeavors. This resource offers nutritious food and fundamental necessities to UC Davis students. Registered students can pick up fresh produce and groceries at The Pantry on campus. In addition, The Pantry collaborates with the Student Health and Counseling Services (SHCS) Teaching Kitchen, a program tailored for college students seeking solutions for healthy eating and sustainable living. The Teaching Kitchen provides students with free cooking demonstrations and samples to emphasize nutrition and culinary skill building. These cooking sessions teach students how to prepare nutritious meals and relay the importance of using seasonal and whole foods when possible. However, students may need more cooking skills and knowledge to use the food resources available at The Pantry effectively. A lack of cooking skills and food preparation has been associated with a decrease in making homemade meals, especially among young adults [11].

To help bridge this gap and offer students more cooking support, take-home meal kits can be beneficial. Take-home meal kits include all the pre-sorted ingredients for an entire meal, which could address potential food waste and help students prepare meals with unfamiliar ingredients [12]. Meal kits provided at food access centers have been proposed as a potential resource to address food insecurity among college students because these kits offer students nutritious food and teach them how to prepare a meal on their own [13]. Other research has showed that meal kits positively improve students’ food security status and increase the likelihood of students returning to these food access centers, which helps to foster a sense of community and belonging [13]. Further, a study recently published in 2021 highlighted that cooking interventions, such as meal kits, can increase college students’ confidence with cooking skills and ultimately contribute to better dietary habits while reducing food insecurity [13]. This research project aims to address food insecurity among college students at UC Davis by developing and investigating the impact of culturally relevant meal kits using accessible food commonly found at the student-run pantry on campus. This exploratory pilot study explores the potential impact of meal kits on students’ cooking self-efficacy, food waste practices, and food security status. The purpose of distributing meal kits is to help reduce food insecurity while fostering culinary skills and reducing food waste. This research also intends to collect insights into the feasibility, acceptability, and potential challenges of integrating meal kits into The Pantry.

## 2. Materials and Methods

### 2.1. Meal Kit Development

A prior needs assessment survey was distributed to UC Davis students in Summer 2023 and Winter 2024 to identify culturally relevant cuisines specific to the student population on campus. The research team subsequently identified six culturally relevant recipes from the UC Davis Teaching Kitchen recipe database that were then adapted to the ingredients mainly available at The Pantry. These recipes were internally tested for any modifications and sampled by UC Davis students in Spring 2024 to collect feedback for any further recipe modifications using a modified sensory evaluation. Of the six recipes, three were selected to be made into take-home meal kits based on high likeability scores. Each recipe represented a different type of cuisine that students wanted from the needs assessment (American, Mexican, or Korean).

**Figure 1:**
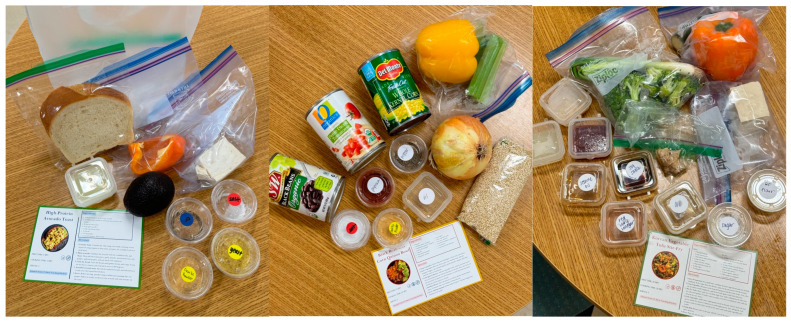
Pre-portioned ingredients for each meal kit, along with the recipe card.

Recipe cards were designed on Canva, an online graphic design tool, for the selected recipes: High-Protein Avocado Toast (American), Mexican-Inspired Quinoa Bowl (Mexican), and a Korean Vegetable Stir Fry (Korean) (Figure S1). All recipes were vegan to ensure that students could consume the meal kits due to possible dietary restrictions. Each recipe card included the preparation/cooking time, serving size, food icons for any allergens, ingredients, and cooking instructions (Figure 1). On the back of the card, information about on-campus food access resources were provided for students, including CalFresh and AggieFresh. CalFresh, also known as SNAP, is a long-term assistance program for low-income people who meet federal eligibility requirements giving them extra financial support to afford healthy and nutritious food [14]. Similarity, AggieFresh is a program that offers eligible students up to USD 292 a month for groceries and restaurant meals, designed for those who do not meet CalFresh’s citizenship or student exemption requirements [15].

### 2.2. Meal Kit Pilot Study Recruitment

Students were invited to participate in the exploratory pilot study by completing a recruitment survey through Qualtrics to determine eligibility. The recruitment survey included questions regarding demographics, access to a kitchen, and willingness to commit to the study parameters (picking up a meal kit or downloading a recipe card once a week for three weeks and completing surveys). Students were eligible to participate if they were 18 years or older, a currently enrolled UC Davis student, and had access to a kitchen. To evaluate the specific impact of the meal kits, a comparison group would only receive a digital recipe card through email. Eligible participants were then randomly selected through CalculatorSoup, a random number generator, to be assigned to the meal kit group (intervention) or recipe card group (comparison).

The recruitment survey was distributed during the recipe sampling pilot test, to students enrolled in an introductory nutrition course (Nutrition 10/10V: Discoveries and Concepts in Nutrition), over UC Davis-related social media accounts, and posted on flyers on campus at Aggie Compass, the Memorial Union, and the Teaching and Learning Center. Students were directed to complete the pre-survey if they meet the eligibility criteria. Eligible participants completed the pre-survey before receiving the first meal kit or recipe card for information about cooking skills, primary food access usage, food waste practices, food security status, and demographics. Students who completed all study measures were given a USD 10 Amazon gift card.

### 2.3. Meal Kit Pilot Study Evaluation

In May 2024, the three meal kits were assembled. All food items were purchased from Costco and/or Trader Joe’s to prevent taking resources from The Pantry. The perishable items, such as green onions and tofu, were purchased a day before the designated prep day to ensure the food would not spoil. The preparation days consisted of portioning the ingredients into the proper containers and packing all containers into the meal kit bag with the corresponding recipe card. All the meal kits were prepared by three ServSafe Food Handler certified research team members at the industrial kitchen at the ASUCD Coffee House (CoHo) on campus under supervision of a food handler manager to ensure proper food safety practices. The meal kits were refrigerated at the CoHo to keep the vegetables and other ingredients fresh for the pickup days. Preparation days were on Mondays followed by a distribution day on Tuesdays, which was when the meal kits were distributed to the study participants. Distribution days were held in the Ragle Human Nutrition Clinical Research on campus. Students were required to sign in with their UC Davis email address to make sure that they were coming to pick up their meal kits. The recipe cards were distributed through email for students who were in the comparison group.

After each meal kit or recipe card was distributed, a feedback survey was emailed out through Qualtrics to collect information about sensory aspects, likelihood of cooking the recipe again, ease of recipe preparation, consumption behaviors, equipment availability, and qualitative feedback. To continue in the study, each participant had to submit the feedback survey before receiving the next meal kit or recipe card. After the last meal kit or recipe card, an 18-item post-survey was emailed through Qualtrics to participants that included the same pre-survey questions with additional questions regarding food waste behaviors and open-ended questions for qualitative feedback. Existing surveys tools were used in the surveys and included the Cooking Self-Efficacy Questionnaire [16] and the 10-item U.S. Adult Food Security Survey Module [17]. Food waste questions were adapted from the International Food Information Council Foundation Consumer Behaviors and Perceptions of Food Waste survey [18]. The Cooking Self-Efficacy Questionnaire assessed participants’ confidence in their ability to prepare meals and follow recipes. A Likert scale was used, where responses ranged as follows: 1 = not at all confident, 2 = slightly confident, 3 = somewhat confident, 4 = fairly confident, 5 = completely confident. Items were summed and a total score was calculated out of 20, with higher scores indicating greater cooking self-efficacy. The 10-item U.S. Adult Food Security Survey Module measured food security by assessing participants’ experiences with food access and availability. Responses of “yes”, “often”, “sometimes”, “almost every month”, and “some months but not every month” were coded as affirmative. Following the scoring procedure, total scores were calculated from 0 to 10, which corresponded to high food security (0), marginal food security (1–2), low food security (3–5), and very low food security (6–10). The food waste questions were adapted from the International Food Information Council Foundation Consumer Behaviors and Perceptions of Food Waste Survey to evaluate participants’ food waste behaviors, including portioning practices and disposal habits. The survey items asked about key components of food waste and response scales varied depending on the specific question.

### 2.4. Data Analysis

Analyses were conducted on data from students that completed all surveys except for the qualitative and sensory feedback for each recipe, which included all submitted responses (Tables S1–S4). Descriptive statistics were expressed as means and SDs for continuous variables and percentages for categorical variables. Categorical variables were calculated into percentages and groups were compared using Fisher’s exact test. The frequency of response for cooking self-efficacy and food waste practices was compared pre- and post-intervention and between the two groups. The change in cooking self-efficacy was calculated by subtracting pre-scores from post-scores. The sum of affirmative responses corresponded to a specified set of items referred to as the household’s raw score. Paired *t* tests were used for pre- and post-intervention comparisons within each group and Student’s *t* tests were used for pre- and post-intervention comparisons between each group for initial exploratory analyses. A 2-way repeated measures ANOVA was used to compare changes in cooking self-efficacy. Stata 16 software (StataCorp, College Station, TX, USA, 2019) was used for all statistical analyses and significance was determined using *p* < 0.05.

Qualitative data from open-ended survey responses were independently coded by two analysts (E.L., M.J.T.) using a structured, numeric coding framework to identify key themes. Each theme was assigned a specific code, which was recorded in a codebook with systematic instructions. An Excel formula was used to flag discrepancies: if the code entered by Coder 1 did not match the code entered by Coder 2, it was marked for review. The analysts discussed discrepancies and reconciled any differences to ensure consistency in theme identification. The frequency of these themes was assessed to compare trends between the intervention and comparison groups. The coding process was conducted manually, with all data entered and tracked using Excel.

## 3. Results

**Figure 2:**
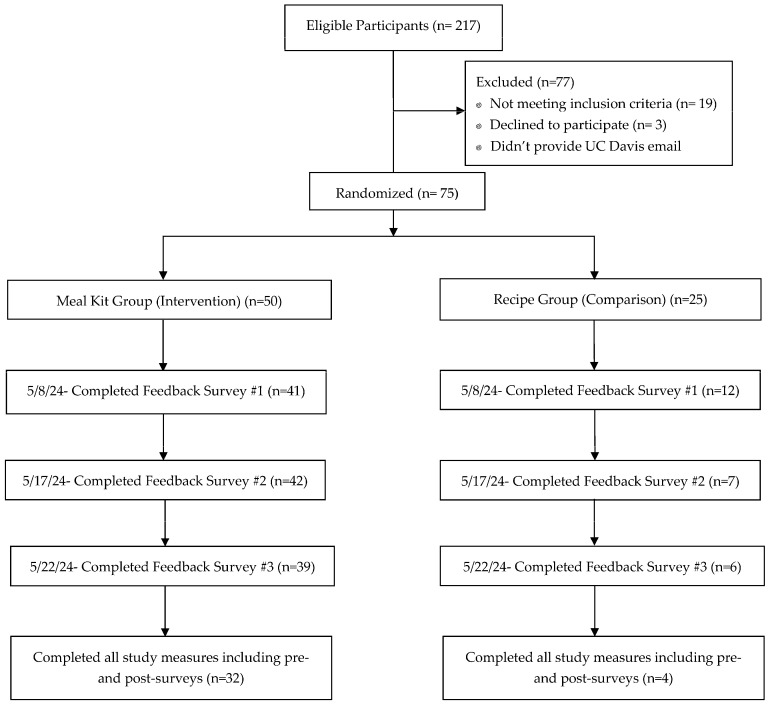
Participant flow from recruitment survey to completion of study measures.

Of the 217 of students who completed the recruitment survey and met the eligibility criteria, 75 participants were randomly selected by a random number generator (Figure 2). As this was an exploratory pilot study, this number was selected due to budgetary and meal kit preparation constraints. Through another random number generator, 50 students were assigned to receive a meal kit (intervention group), while 25 students were assigned to receive recipe cards (comparison group). In total, 42 in the intervention group and 19 in the comparison group agreed to participate. One participant in the intervention group was excluded from the analysis for not completing the pre-survey. Over the course of the 3-week exploratory pilot study, 32 in the intervention group and 4 in the comparison group completed all study measures.

Most participants identified as female, Asian, and were sophomore or juniors in college (Table 1). The average age of the participants was 20 years. Additionally, most participants were not currently on a university meal plan. Regardless of intervention group assignment, half of the participants reported using the ASUCD Pantry on campus (Table 1). There were no significant differences in baseline characteristics between groups.

### 3.1. Food Security Status

From the pre-survey, 75% of the comparison group were classified as having low food security status and 25% were classified as having very low security status, whereas 50% of the participants in the intervention group were classified as having high food security (Table 2). After the 3-week intervention, both groups showed an improvement in food security status: 75% of the comparison group and 63% of the intervention group were classified as having high food security status. However, this improvement was only significant in the meal kit group (*p* < 0.01) and not significant in the recipe group (*p* = 0.25).

### 3.2. Cooking Self-Efficacy

Participants in the meal kit group (intervention) experienced a significant 1.2-point increase in overall cooking self-efficacy (*p* < 0.01), while the recipe group (comparison) had a 1.5-point deduction (*p* = 0.52; Table S1). When comparing the change in cooking self-efficacy between groups, there was a −2.7-point difference (*p* = 0.07; Table 3). For the intervention group, there was an increase from 28% to 31% of participants who were “completely confident” in cooking a nutritious meal and an increase from 44% to 53% of participants who were “fairly confident”. Similar trends were observed in cooking a meal in a short time, where “fairly confident” participants increased from 31% to 50%. Further, the confidence in cooking a nutritious meal on a budget increased from 22% to 50% for “fairly confident” (Table S1). Although the recipe group (comparison) had a smaller sample size, complete confidence in cooking a nutritious meal increased from 50% to 75%. However, when examining differences in each scale item for cooking self-efficacy between groups, there were no significant differences (Table 3).

### 3.3. Food Waste Outcomes

From pre- to post-, the meal kit group (intervention) reported changes in food waste, such as slightly more waste from meal leftovers from home and fresh produce, whereas there was a reduction in waste from restaurant leftovers and cheese/yogurt (Table 4). Participants also showed a heightened awareness of food waste during grocery shopping and dining out. The intervention group also thought about food waste more often while eating out and eating at home. For the recipe group (comparison), there were a decrease in specific food waste, such as restaurant leftovers and animal products. When asked about food waste while grocery shopping, there was a decrease in concerns about reducing money spent and an increase in reducing waste in all aspects (Table 4). The recipe group also shifted from “always” having food waste on their minds to “sometimes”.

### 3.4. Feedback for Meal Kits and Recipes

For the High-Protein Avocado Toast (Recipe #1), the meal kit group showed high engagement, with 93% of participants cooking the recipe and 95% following it as intended (Table S2). Most of the participants (61%) consumed the entire meal and reported the meal kit made one to two meals. About 41% had leftovers and 85% of participants consumed all the ingredients from the meal kit. In total, 83% of participants enjoyed the recipe, 98% had the necessary equipment, and 85% found the recipe easy to make. Similarly, in the recipe group, 92% made the recipe and 75% followed it as intended. Sixty-seven percent consumed the entire meal and half of the participants found it very easy to make.

For the Korean Vegetable Tofu Stir Fry (Recipe #2) and the Mexican-Inspired Quinoa Bowl (Recipe #3), 100% participants from the meal kit groups cooked the recipe and nearly all followed the recipe as intended (Tables S3 and S4). In both recipes, the majority consumed a significant portion of the recipe, found it easy to make, and had the necessary equipment. There were high leftover rates (60% for the stir-fry and 76% for the quinoa bowl), indicating multiple meals. The enjoyment was high for both recipes (78% for the stir-fry and 82% for the quinoa bowl). The recipe group also showed strong engagement with high satisfaction and reported the Mexican-Inspired Quinoa Bowl recipe had high leftover rates.

Several themes emerged from the qualitative data collected on each feedback survey, highlighting the participants’ experiences and perceptions. These included ease of preparation, ingredient familiarity and accessibility, cooking skills and equipment, recipe modifications, and overall impressions (Table S5).

#### 3.4.1. Ease of Preparation

Participants appreciated the simplicity of the meal kits, describing how the instructions were clear, and the steps were easy to follow. Recipes were especially recognized for their minimal preparation, even among individuals with limited cooking experience. While many found the process quick and efficient, certain aspects, such as cooking the quinoa or chopping the various vegetables, were noted as time-consuming. These tasks, while straightforward for some, highlighted varying levels of cooking skills with meal preparation among participants.

#### 3.4.2. Ingredient Familiarity and Accessibility

The ingredients provided in the meal kits were generally well-received, as they often included familiar items, such as avocados, tofu, and bell peppers. The pre-portioned ingredients were noted for reducing food waste and simplifying the participant’s cooking experience. However, less common ingredients, like nutritional yeast in Recipe #1 and fresh ginger in Recipe #2, received mixed reactions. For some participants, these ingredients were an exciting opportunity to try new foods, while others found them less appealing or challenging to incorporate into their usual diets.

#### 3.4.3. Cooking Skills and Equipment

The meal kits were designed to cater to a range of cooking abilities, yet some participants encountered challenges due to limited skills or a lack of necessary kitchen tools. For example, handling tofu, cutting vegetables like onions and bell peppers, and cooking quinoa were common struggles. Some participants also mentioned difficulties with using certain equipment, such as a broken can opener or lacking a large pot, which made it harder to follow the recipes accurately. These challenges highlighted the varying levels of kitchen proficiency and the need for more accessible guidance on basic cooking techniques and tool requirements.

#### 3.4.4. Recipe Modifications

Recipe modifications were a consistent theme, with many participants adapting the recipes to align with their personal dietary preferences. Popular modifications included adding spices, like chili oil or soy sauce, to enhance flavors, or incorporating other ingredients, such as avocado, cheese, or tortilla chips. Interestingly, those who modified the recipes generally had a more positive outlook on the meal kits. Some recipe cards suggested modifications, such as substituting ingredients with meat or adding avocado, which allowed participants to tailor the recipe to their liking and contributed to a more favorable experience.

#### 3.4.5. General Perceptions

Overall, participants responded positively to the meal kits, emphasizing their convenience, affordability, and role in encouraging nutritious eating. Many appreciated the suitability of the kits for meal prepping due to the large portions provided. Despite this, several negative comments were noted. Concerns about the environmental impact of packaging waste and questions regarding the safe storage of partially used ingredients, such as tofu or avocados, were recurring points of feedback.

## 4. Discussion

This exploratory pilot study aims to address food insecurity among UC Davis college students by investigating the impact of culturally relevant meal kits using ingredients from the on-campus student-run pantry. Preliminary results from the exploratory pilot study showed that after use of the meal kits or recipe cards for three weeks, both groups experienced an improvement in food security classification, with 63% of the intervention group and 75% of the comparison group classified as having high food security status. Although not a significant finding between groups, there was a trend of improved cooking self-efficacy, particularly in the intervention group, who showed an increase in confidence levels regarding cooking nutritious meals on a budget.

The intervention group also had more awareness about food waste, primarily focusing on cost reduction, while the comparison group demonstrated a more balanced approach to waste reduction, considering factors such as environmental impact, overall food waste, and cost effectiveness. This suggests a broader understanding of waste, reflecting a shift beyond just financial concerns. However, it is important to note that even though these participants might feel motivated to reduce food waste during this study, they may continue their pre-existing food reduction practices after the intervention because of established habits. Additionally, participation may have been motivated by the financial incentive rather than personal goals [19]. Interestingly, there was a reduction in waste from restaurant leftovers across the groups, which may suggest that inclusion of the meal kits and recipe cards encouraged more cooking at home. However, there was a slight increase in waste from meals prepared at home in the intervention group, which may have been due to larger-than-expected portions or participants excluding ingredients they were unfamiliar with or did not prefer. Additionally, some participants expressed concerns about how to store leftovers properly and were unsure of food safety protocols, which may have furthered contributed to food waste. Future work can consider offering meal kits with different portion sizes, as well as variation in ingredients to accommodate tastes/preferences to help reduce food waste. Including information about proper storage and food safety practices can also potentially help to reduce food waste.

The preliminary findings from this exploratory pilot study align with previous research that found culturally tailored interventions can help to improve food-related behaviors and outcomes, particularly among racial and ethnic minority groups by addressing cultural preferences and barriers [20]. These preliminary findings are further supported by research conducted on a semester-long food skills course with a teaching kitchen, which found that hands-on cooking interventions improved cooking self-efficacy, cooking frequency, and reduced meal-skipping among college students [10]. Similar to this intervention, the culturally relevant meal kits in our study empowered students to develop confidence in preparing affordable, nutritious meals while fostering greater awareness about food security and waste reduction. Both interventions demonstrate the value of hands-on learning approaches in overcoming challenges, such as limited resources and food literacy, to foster healthier eating habits [10]. Similarly, a study conducted by Truong et al., in 2014, highlights the importance of culturally relevant interventions to help increase engagement and effectiveness, which helps to support the positive feedback and high satisfaction rates seen from the study participants [21]. These parallels suggest that integrating culturally relevant meal kits into college campuses could be an effective strategy for improving food security and promoting healthier eating habits among this population. Additionally, findings from Martinez et al. highlight that college food pantry use is associated with self-reported improvements in sleep, mental health, and overall physical health. This study showed that increased visits to the campus food pantries were directly associated with decreased depressive symptoms and improved perceived health and sleep sufficiency. These findings further reinforce the notion that providing college students with accessible and nutritious food resources, such as meal kits or food pantries, can yield benefits beyond food security, potentially contributing to mental and physical well-being [22].

This exploratory pilot aligns with existing knowledge and public health context by addressing food insecurity among college students, which is a well-known issue with significant health and academic outcomes. Previous studies have highlighted the prevalence of food insecurity in this population and its association with poor eating habits and mental health concerns [23]. In 2021, a randomized intervention study conducted by the University of Vermont implemented six weeks of cooking classes for college students and six weekly home-delivered meal kits for college students aged 18–22. The participants that were part of the meal kit intervention group had a significant improvement in their food agency, as well as how they planned, prepared, and procured food as students [13]. In terms of the broader public health context, this project contributes to the Healthy People 2030 goals, which aim to improve food security and reduce disparities in food access. By exploring interventions, such as culturally relevant meal kits, this project seeks to enhance food security and help to promote healthier eating habits among college students. Further, this intervention was in partnership with other campus organizations, including The Pantry, the Teaching Kitchen, the CoHo, and the Ragle Human Nutrition Center. Collaborating with existing organizations can contribute to sustainable programming and help improve student’s food security. A pilot study conducted by the University of California, Berkeley, found that including a teaching kitchen within a college-level nutrition class can help increase food security status among students [24]. This strategy is in line with the Centers for Disease Control recommendations for increasing implementation in holistic food security programs that help to maximize community resources and partnerships to cultivate long-lasting positive changes.

This project demonstrates that culturally relevant meal kits can effectively address food security among college students by improving access to nutritious foods and enhancing cooking skills. This can contribute to improved physical and mental health, which can help with academic performance. However, the dramatic increase in food security classification from the comparison group was unexpected. This shift may have been influenced by external factors not captured by study measures, such as the seasonality of food security and the use of additional resources/support. Thus, for future research it would be important to include focus groups or interviews at the conclusion of the intervention to capture a more in-depth understanding of the complex influences on food security status [25]. Changes in financial aid disbursements, access to off-campus food resources, and changes in employment status are additional factors that have been identified as critical determinants of food security among college students [25]. Altogether, the project’s promise in improving food security status, food waste practices, and cooking self-efficacy suggests that similar interventions on other college campuses can be beneficial to help reduce the food security rates. Moreover, the study addresses the importance of integrating culturally relevant preferred foods into food security-related programs to improve the utilization and effectiveness of these resources. The results of this study are consistent with previous research that highlight the effectiveness of hands-on cooking programs in improving food security among college students. For instance, another study done at the University of California, Berkeley in 2021 found that adding culinary education to university courses helped to boost food security and reduce stress. Similarly, our study showed improvements in food security and cooking confidence, especially among the intervention group. Together, these findings suggest that culturally relevant cooking programs/interventions can be a vital tool for addressing food insecurity and promoting healthier habits [26]. By collaborating with existing college campus food access resources, like The Pantry and the Teaching Kitchen, the project emphasizes the value of including community partnerships to help collectively reduce food insecurity. The feedback forms reflected high engagement and satisfaction rates among participants, especially in following the three recipes, consuming the meals, and finding preparing the meals easy. Collectively, these themes reveal a balanced view of the meal kits. While the accessibility and ease of the meal kits were strong positives, challenges, like unclear instructions, flavor inconsistencies, and ingredient management, suggest areas for future improvement. Incorporating more detailed guidance on preparation and storage, either in a video format or by offering modification options, may further enhance the meal kits’ appeal and usability.

One of the study’s primary strengths is its focus on culturally relevant foods to target different cuisines that students wanted foods from on campus. Additionally, the recipes catered to the diverse dietary preferences of the student population, such as creating vegetarian recipes and removing most major allergens. Prior to the exploratory pilot study, the recipes were tested internally and subsequently sampled by UC Davis students to gather specific feedback to modify the recipes before meal kit implementation. This helped to ensure the meal kits would be well-received and easy for students to prepare at home. The study included pre- and post-surveys to assess cooking self-efficacy, food waste practices, and food security status, as well as a feedback survey for each meal kit or recipe. These comprehensive data being collected allowed for a thorough analysis of the intervention’s impact. Despite the short duration of the study, participants had a high level of engagement and responsiveness. The quick turnaround in data collection reflected a strong interest in the meal kits, indicating the intervention’s appeal and relevance to this student population. The three-week duration of this meal kit intervention aligns with previous studies that have conducted similar studies over a short duration and demonstrated meaningful impacts on dietary behaviors. Hollis-Hansen et al. (2023) conducted a two-week randomized controlled trial that compared meal kits to no-prep meals among food pantry clients and found significant improvements in perceived diet quality and food security [27]. Similarly, Robinson-Oghogho et al. (2023) implemented an eight-week meal kit intervention and found increased vegetable consumption and improved meal preparation behaviors [28]. Additionally, Mialki et al. (2020) conducted a six-week meal kit intervention among low-income families and reported high adherence rates and participant satisfaction [29]. Despite the short intervention periods in these studies, these findings suggest that meal kit interventions can effectively influence dietary behaviors, which supports the feasibility of the three-week timeframe used in this exploratory pilot study.

A major limitation of this study is the small sample size, particularly in the recipe group (comparison group). The small number of participants in the comparison group prevents the detection of notable significant differences between the groups for this exploratory work. Due to budgetary constraints, the research team was unable to include more participants at the beginning of the study; however, for future work it would be imperative to include an initial, larger sample, especially for participants in the comparison group. Incorporating additional resources and education about food literacy may also help to improve outcomes related to cooking skills, food waste, and food security classification. Future work should consider supplemental handouts and/or video tutorials about food literacy topics, such as proper leftover storage and basic cooking skills (e.g., knife skills). Another challenge was the loss to follow-up, particularly in the comparison group, since the number of participants decreased over the duration of the study. This can be addressed by implementing a crossover design so that participants in the comparison group can have the opportunity to receive meal kits after the first phase. Including a greater incentive for participation in addition to the USD 10 gift card could also be considered, such as cooking equipment, like pots and pans, since some participants expressed that they did not have all of the necessary cooking supplies (Table S5). Moreover, the study’s duration was only three weeks. This might not be enough time to observe significant long-term changes. A longer intervention period would provide more insights to see whether there are lasting significant differences. Lastly, it was challenging to identify partners on campus who would be able to assist with preparing foods in a food-safe-certified commercial kitchen, leading to a delay in study implementation. For future work, it would be helpful to have a campus guide for how to distribute food to students following food safety protocols to allow for more projects like this one.

### Recommendations for Future Work

Based on the findings of this exploratory pilot study, several recommendations can be made for future work. First, it is important to increase the sample size for both groups, especially in the comparison group, to enhance the generalizability of the study. A larger and more diverse sample can provide greater insight for identifying significant differences and patterns. To add on, extending the study duration to more than three weeks would allow for the observation of long-term changes in cooking self-efficacy, food security status, and food waste practices, which would offer more insights into the impact of this intervention. To help improve study retention, creating a Google phone number or Discord server could help to inform students when to complete all the feedback forms, since students may not check their school email often. For the comparison group, additional incentives are needed to retain study participation. It could be considered to offer grocery store vouchers, so they could purchase the ingredients needed for each recipe, or to employ a delayed intervention, so that the group would receive meal kits at the conclusion of the study for added incentive. Although the feedback for the meal kits was positive, the concerns about the environmental impact of packaging could be addressed by using reusable and/or recyclable material. Including instructions about how to store leftovers and partially used ingredients could also be beneficial. Additionally, having supplemental videos that show the recipe preparation process, which includes specific cooking techniques, food safety principles, and the final form of the meal, could help to bolster the improvement seen in cooking self-efficacy. Further, strengthening communication and coordination among campus food access resource organizations can lead to more collaboration and lasting partnerships. Developing standard protocols regarding distributing food to students with clear guidelines can streamline the collaboration process with other programs at UC Davis. Lastly, with the recipe feedback data from this study, campus organizations, such Aggie Eats and the CoHo, can potentially adapt these recipes to provide to students, while The Pantry can possibly adapt this meal kit intervention and disseminate meal kits to students as part of their programming.

## 5. Conclusions

The results from this exploratory pilot showed that the meal kit and recipe group experienced an increase in food security status over the three-week intervention period, with 63% of the intervention group and 75% of the comparison group being classified as having high food security status. Further, the intervention group significantly increased cooking confidence, specifically in preparing nutritious meals on a budget and within a short time. Food waste practices also improved in the intervention group, who reported focusing more on reducing food costs, while the recipe group focused on considering environmental and food cost factors. The meal kits and recipes were well-received by participants, with the majority consuming the meals and reporting high enjoyment. Several themes emerged from the qualitative data, including ease of preparation and familiarity with ingredients. Participants appreciated that there was room to modify the recipes to cater to their personal dietary preferences, as well as the minimal cooking preparation involved. Although most feedback was positive, there were some concerns about the environmental impact of the packaging waste, as well as how to store partially used ingredients. This could be addressed through reusable and/or recyclable materials, as well as supplemental videos addressing the recipe preparation process. This exploratory pilot study highlights the importance of culturally relevant interventions to address food security and promote healthier eating habits among college students. Additionally, when providing recipes to participants, it may be helpful to encourage modifications to their liking for greater enjoyment. The findings from this research underscore the potential for meal kits to serve as an effective tool in reducing food insecurity and fostering a better sense of community and campus resource utilization.

## Figures and Tables

**Table 1:** Demographics of participants.

Characteristic		Meal Kit Group (n = 32)	Recipe Group (n = 4)	*p*-Value
Sex, n (%)	Female	29 (90)	3 (75)	0.39
	Male	3 (9)	1 (25)	
Age in years, mean (SD)		20.50 (1.48)	20.0 (1.40)	
Race/Ethnicity, n (%)	African American/Black, not of Hispanic origin	2 (6)	0 (0)	0.15
	American Indian/Alaska native	0 (0)	0 (0)	
	Middle Eastern/North African	1 (3)	0 (0)	
	Asian	20 (63)	1 (25)	
	White, not of Hispanic origin	4 (13)	1 (25)	
	Latino/a/x/Chicano/a/x/Hispanic (Mexican American, Puerto Rican, Cuban)	2 (6)	1 (25)	
	Multiple reported	3 (9)	1 (25)	
Enrollment status	UC Freshman (1st-year)	4 (13)	0 (0)	0.71
	UC Sophomore (2nd-year)	9 (28)	2 (50)	
	UC Junior (3rd-year)	9 (28)	2 (50)	
	UC Senior (4th-year)	8 (25)	0 (0)	
	UC 5th-year	2 (6)	0 (0)	
Transfer student status, n (%)	Yes	6 (19)	0 (0)	1.0
	No	25 (78)	4 (100)	
	Unreported	1 (3)	0 (0)	
International student status, n (%)	Yes	3 (9)	0 (0)	1.0
	No	28 (88)	4 (100)	
	Unreported	1 (3)	0 (0)	
Student parent status, n (%)	Yes	0 (0)	0 (0)	1.0
	No	32 (100)	4 (100)	
Do you have a job in addition to being a student?, n (%)	Yes	21 (66)	2 (50)	0.83
	No	10 (31)	2 (50)	
	Unreported	1 (3)	0 (0)	
Currently participating in a meal plan?, n (%)	Yes	2 (6)	0 (0)	0.79
	No	30 (94)	4 (100)	
Do you use The ASUCD Pantry on the UC Davis campus?, n (%)	Yes	16 (50)	2 (50)	0.70
	No	16 (50)	2 (50)	

**Table 2:** Food security status based on the 10-item U.S. Adult Food Security Survey Module of participants pre- and post-measure.

Food Security Classification, n (%)	Meal Kit Group (n = 32)		Recipe Group (n = 4)	
	Pre-	Post-	Pre-	Post-
High Food Security	16 (50)	20 (63)	0 (0)	3 (75)
Marginal Food Security	6 (19)	2 (6)	0 (0)	0 (0)
Low Food Security	7 (22)	4 (12)	3 (75)	1 (25)
Very Low Food Security	3 (9)	6 (19)	1 (25)	0 (0)

**Table 3:** Comparison of cooking self-efficacy pre- and post-measure using repeated ANOVA.

Questions, Mean (SD)	Meal Kit Group (n = 32)		Recipe Group (n = 4)		*p*-Value
	Pre-	Post-	Pre-	Post-	
How confident are you in being able to cook a nutritious meal?	3.91 (0.93)	4.09 (0.82)	4.00 (0.58)	4.25 (1.50)	0.42
How confident are you in being able to cook a meal in a short period of time?	3.50 (1.02)	3.72 (0.85)	4.25 (0.82)	3.5 (1.00)	0.27
How confident are you in being able to cook a nutritious meal on a budget?	3.12 (0.90)	3.62 (0.90)	4.25 (0.50)	3.75 (0.50)	0.06
How confident are you in being able to follow a recipe?	4.38 (0.87)	4.69 (0.47)	4.50 (0.58)	4.25 (0.96)	0.17
Summative Score (max = 20 points)	14.9 (2.8)	16.1 (2.4)	17.2 (2.2)	15.8 (2.4)	0.07

**Table 4:** Comparison of food waste outcomes pre- and post- measure.

	Meal Kit Group (n = 32)		Recipe Group (n = 4)	
	Pre-	Post-	Pre-	Post-
Thinking back over the last month, what types of foods most often end up in your garbage at home? Select all that apply.				
Meal leftover from home, n (%)	17 (53)	19 (59)	2 (50)	3 (75)
Fresh produce, n (%)	17 (53)	19 (59)	2 (50)	2 (50)
Meal leftover from restaurants, n (%)	7 (22)	3 (9)	2 (50)	0 (0)
Animal products, n (%)	2 (6)	4 (13)	0 (0)	0 (0)
Milk, n (%)	9 (28)	9 (28)	0 (0)	0 (0)
Shelf-stable items, n (%)	1 (3)	3 (9)	2 (50)	0 (0)
Cheese or yogurt, n (%)	8 (25)	4 (13)	0 (0)	1 (25)
Milk alternatives, n (%)	2 (6)	1 (3)	0 (0)	0 (0)
Other, n (%)	3 (9)	1 (3)	0 (0)	0 (0)
Why do these foods end up in your garbage? Please select your top two reasons.				
It spoiled or became stale, n (%)	28 (88)	28 (88)	2 (50)	2 (50)
I was cleaning out the refrigerator/pantry, n (%)	11 (34)	12 (38)	3 (75)	1 (25)
Others in my household didn’t want to eat it, n (%)	4 (13)	6 (18)	2 (50)	2 (50)
I didn’t want to eat it, n (%)	9 (28)	9 (28)	0 (0)	1 (25)
It was more food than I wanted to eat, n (%)	1 (3)	5 (16)	2 (50)	3 (75)
While grocery shopping, how often is food waste (food that gets thrown away) on your mind?				
Always, n (%)	15 (47)	15 (47)	2 (50)	0 (0)
Sometimes, n (%)	15 (47)	15 (47)	2 (50)	4 (100)
Never, n (%)	2 (6)	2 (6)	0 (0)	0 (0)
While eating out, how often is food waste (food that gets thrown away) on your mind?				
Always, n (%)	11 (34)	13 (41)	1 (25)	1 (25)
Sometimes, n (%)	16 (50)	13 (41)	2 (50)	2 (50)
Never, n (%)	5 (16)	6 (18)	1 (25)	1 (25)
While eating at home, how often is food waste (food that gets thrown away) on your mind?				
Always, n (%)	12 (38)	14 (44)	3 (75)	1 (25)
Sometimes, n (%)	18 (56)	16 (50)	1 (25)	3 (75)
Never, n (%)	2 (6)	2 (6)	0 (0)	0 (0)
What is the top reason food waste is on your mind while grocery shopping?				
Reduce amount of money spent, n (%)	12 (38)	16 (50)	2 (50)	0 (0)
Reduce waste in all aspects, n (%)	14 (44)	11 (34)	1 (25)	4 (100)
Concerned about environment, n (%)	0 (0)	0 (0)	0 (0)	0 (0)
Ensuring food availability, n (%)	4 (13)	2 (6)	1 (25)	0 (0)
Follow long-time habits, n (%)	2 (6)	3 (9)	0 (0)	0 (0)
What is the top reason food waste is on your mind eating out?				
Reduce amount of money spent, n (%)	12 (38)	14 (44)	2 (50)	2 (50)
Reduce waste in all aspects, n (%)	12 (38)	10 (31)	1 (25)	2 (50)
Concerned about environment, n (%)	1 (3)	0 (0)	0 (0)	0 (0)
Ensuring food availability, n (%)	3 (9)	2 (6)	1 (25)	0 (0)
Follow long-time habits, n (%)	4 (13)	6 (19)	0 (0)	0 (0)
What is the top reason food waste is on your mind while eating at home?				
Reduce amount of money spent, n (%)	6 (19)	4 (13)	0 (0)	0 (0)
Reduce waste in all aspects, n (%)	16 (50)	15 (47)	2 (50)	3 (75)
Concerned about environment, n (%)	1 (3)	1 (3)	0 (0)	1 (25)
Ensuring food availability, n (%)	5 (16)	6 (19)	2 (50)	0 (0)
Follow long-time habits, n (%)	4 (13)	6 (19)	0 (0)	0 (0)
In what ways do you try to reduce the amount of food waste in your home? Please select all that apply.				
Store to maximize shelf life, n (%)	20 (63)	23 (72)	3 (75)	3 (75)
Keep organized pantry, n (%)	18 (56)	23 (72)	3 (75)	3 (75)
Make grocery list, n (%)	20 (63)	22 (69)	4 (100)	3 (75)
Meal plan, n (%)	10 (31)	17 (53)	3 (75)	3 (75)
Compost, n (%)	10 (31)	6 (19)	2 (50)	0 (0)
Shop for groceries online, n (%)	4 (13)	4 (13)	1 (25)	1 (25)
Donate food, n (%)	4 (13)	4 (13)	1 (25)	2 (50)
Other, n (%)	3 (9)	2 (6)	1 (25)	0 (0)
None, n (%)	0 (0)	0 (0)	0 (0)	0 (0)
In what ways do you try to reduce the amount of food waste while eating out? Please select all that apply.				
Take leftovers home, n (%)	32 (100)	32 (100)	4 (100)	4 (100)
Order small meal, n (%)	17 (53)	20 (63)	3 (75)	3 (75)
Share meals, n (%)	22 (69)	26 (82)	3 (75)	1 (25)
Personalize meals, n (%)	7 (22)	5 (16)	1 (25)	2 (50)
Other, n (%)	3 (9)	0 (0)	0 (0)	0 (0)
None, n (%)	0 (0)	0 (0)	0 (0)	0 (0)

## Data Availability

Data available upon request.

## Supplementary Materials

The following supporting information can be downloaded at: https://www.mdpi.com/article/10.3390/nu17050843/s1, Figure S1. Recipe cards distributed physically in the meal kits (meal kit group) or virtually online (recipe card group). Table S1. Comparison of cooking self-efficacy pre- and post-measure. Table S2. Feedback for High-Protein Avocado Toast (recipe #1). Table S3. Feedback for the Korean Vegetable Tofu Stir Fry (recipe #2). Table S4. Feedback for the Mexican-Inspired Quinoa Bowl (recipe #3). Table S5: Qualitative feedback from participants.

## References

[1] Martinez S.M., Frongillo E.A., Leung C., Ritchie L. (2020). No food for thought: Food insecurity is related to poor mental health and lower academic performance among students in California’s public university system. J. Health Psychol..

[2] Richmond C., Steckley M., Neufeld H., Kerr R.B., Wilson K., Dokis B. (2020). First Nations Food Environments: Exploring the Role of Place, Income, and Social Connection. Curr. Dev. Nutr..

[3] Seligman H.K., Laraia B.A., Kushel M.B. (2010). Food Insecurity Is Associated with Chronic Disease among Low-Income NHANES Participants. J. Nutr..

[4] Hagedorn-Hatfield R.L., Hood L.B., Hege A. (2022). A Decade of College Student Hunger: What We Know and Where We Need to Go. Front. Public Health.

[5] Abraham S., Noriega B.R., Shin J.Y. (2018). College students’ eating habits and knowledge of nutritional requirements. J. Nutr. Hum. Health.

[6] Mengi Çelik Ö., Aytekin Şahin G., Gürel S. (2023). Do cooking and food preparation skills affect healthy eating in college students?. Food Sci. Nutr..

[7] U.S. Department of Agriculture and U.S. Department of Health and Human Services (2020). Dietary Guidelines for Americans, 2020–2025.

[8] Gramza-Michałowska A. (2020). The Effects of Ultra-Processed Food Consumption—Is There Any Action Needed?. Nutrients.

[9] Phillips E., McDaniel A., Croft A. (2018). Food Insecurity and Academic Disruption Among College Students. J. Stud. Aff. Res. Pract..

[10] French C.D., Gomez-Lara A., Hee A., Shankar A., Song N., Campos M., McCoin M., Matias S. (2024). Impact of a Food Skills Course with a Teaching Kitchen on Dietary and Cooking Self-Efficacy and Behaviors among College Students. Nutrients.

[11] Reicks M., Trofholz A.C., Stang J.S., Laska M.N. (2014). Impact of Cooking and Home Food Preparation Interventions Among Adults: Outcomes and Implications for Future Programs. J. Nutr. Educ. Behav..

[12] Schuster S., Speck M., Van Herpen E., Bos-Brouwers H. (2022). Do meal boxes reduce food waste from households?. J. Clean. Prod..

[13] Pope L., Alpaugh M., Trubek A., Skelly J., Harvey J. (2021). Beyond Ramen: Investigating Methods to Improve Food Agency among College Students. Nutrients.

[14] CalFresh Program. https://www.cdss.ca.gov/calfresh.

[15] Aggiefresh, Aggie Compass. https://aggiecompass.ucdavis.edu/aggiefresh.

[16] Gaines A., Robb C.A., Knol L.L., Sickler S. (2014). Food Security and Resource Adequacy. Int. J. Consum. Stud..

[17] U.S. Department of Agriculture, Economic Service Research US Household Food Security Survey Module. https://www.ers.usda.gov/topics/food-nutrition-assistance/food-security-in-the-us/survey-tools/#household.

[18] IFIC Foundation A Survey of Consumer Behaviors and Perceptions of Food Waste. https://foodinsight.org/wp-content/uploads/2019/09/IFIC-EPAL-Food-Waste-Deck-Final-9.16.19.pdf.

[19] Soma T., Li B., Maclaren V. (2021). An Evaluation of a Consumer Food Waste Awareness Campaign Using the Motivation Opportunity Ability Framework. Resour. Conserv. Recycl..

[20] Joo J.Y., Liu M.F. (2021). Culturally tailored interventions for ethnic minorities: A scoping review. Nurs. Open.

[21] Truong M., Paradies Y., Priest N. (2014). Interventions to improve cultural competency in healthcare: A systematic review of reviews. BMC Health Serv. Res..

[22] Martinez S.M., Chodur G.M., Esaryk E.E., Kaladijian S., Ritchie L.D., Grandner M. (2022). Campus Food Pantry Use Is Linked to Better Health Among Public University Students. J. Nutr. Educ. Behav..

[23] Bruening M., Van Woerden I., Todd M., Laska M.N. (2018). Hungry to learn: The prevalence and effects of food insecurity on health behaviors and outcomes over time among a diverse sample of university freshmen. Int. J. Behav. Nutr. Phys. Act..

[24] Matias S.L., Rodriguez-Jordan J., McCoin M. (2020). Utilization of a Teaching Kitchen Within a Nutrition Course to Reduce Food Insecurity Among College Students. J. Nutr. Educ. Behav..

[25] Sklar E., Chodur G.M., Kemp L., Fetter D.S., Scherr R.E. (2025). Food Acquisition Coping Strategies Vary Based on Food Security Among University Students. Curr. Dev. Nutr..

[26] Matias S.L., Rodriguez-Jordan J., McCoin M. (2021). Integrated Nutrition and Culinary Education in Response to Food Insecurity in a Public University. Nutrients.

[27] Hollis-Hansen K., Haskins C., Turcios J., Bowen M.E., Leonard T., Lee M., Albin J., Wadkins-Chambers B., Thompson C., Hall T. (2023). A Pilot Randomized Controlled Trial Comparing Nutritious Meal Kits and No-Prep Meals to Improve Food Security and Diet Quality Among Food Pantry Clients. BMC Public Health.

[28] Robinson-Oghogho J.N., Palmer A., Davey-Rothwell M., Thorpe R.J. (2023). Evaluating a Washington DC Community-Based Meal-Kit Service Aimed at Mitigating Dietary Disparities: Results from the SouthEats Pilot Study. Prev. Med. Rep..

[29] Mialki K., Sweeney L., House L., Shelnutt K. (2020). O13 Acceptability and Affordability of a Meal Kit Intervention for Low-Income Families. J. Nutr. Educ. Behav..

