# Supplementary material for: Using Culturally Relevant Meal Kits to Improve Cooking Skills, Reduce Food Waste, and Promote Engagement with a Campus Food Access Resource: An Exploratory Pilot Study
Source: Nutrients. 2025 Feb 28;17(5):843. doi: 10.3390/nu17050843 (PMC11901532; doi:10.3390/nu17050843)
Supplement: Supplementary file 1 [file nutrients-17-00843-s001.zip › nutrients-3471101-supplementary.pdf]

Figure S1: Recipe cards distributed physically in the meal kits (meal kit group) or virtually online (recipe card group).

High-Protein Avocado Toast

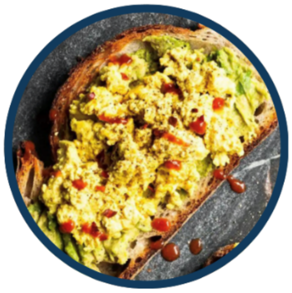

PREP TIME: 5 MIN

COOKING TIME: 5 MIN

SERVES: 4

Adapted from UC Davis Teaching Kitchen

Ingredients

- 1 small avocado or ½ of a large avocado
- 1/4 block tofu crumbled and liquid squeezed out with hands
- 2 tbsp of lemon juice
- ¾ tsp garlic powder
- 1 tbsp nutritional yeast
- ¼ tsp salt
- 1/8 tsp pepper
- ¼ red bell pepper, finely diced

Directions

1. **Crumble Tofu:** Crumble the tofu using your hands, draining excess liquid (if using regular or firm tofu) and place the crumbled tofu in a small bowl.
2. **Mash avocado:** Combine the avocado with the crumbled tofu, and Mash. Then add the lemon juice, garlic powder, nutritional yeast, salt, pepper, and bell pepper, and mix until well combined.
3. **Toast the bread:** Toast the bread until golden brown and crispy. If you do not have a toaster, place bread in oven at 350˚degrees.
4. **Assemble avocado toast:** Spread the mashed avocado and tofu mixture evenly over the toasted bread slices.
5. **Serve:** Before serving, sprinkle desired additional toppings like red pepper flakes or sesame seeds over the avocado and tofu mixture on the toast.

Korean Vegetable Tofu Stir-Fry

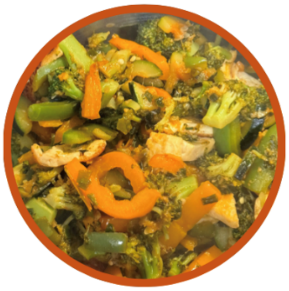

PREP TIME: 25 MIN

COOKING TIME: 32 MIN

SERVES: 4

Adapted from UC Davis Teaching Kitchen

Ingredients

Sauce

- 2 tbsp gochujang
- 2 tbsp lemon juice
- 1 tbsp finely minced fresh ginger
- 1 tbsp soy sauce
- 1 tbsp red wine vinegar
- 1 tbsp toasted sesame oil
- 2 tbsp sugar
- 1½ tbsp gluten free flour

Stir-Fry

- 3 tbsp cooking oil, divided
- ½ block of 14 oz extra firm block tofu, cubed
- 4 cups 1-inch broccoli florets
- 1 bunch green onions, chopped into ½ inch pieces
- 4 cloves garlic, minced
- 2 bell peppers,chopped
- 1 zucchini, chopped

Directions

1. **Sauce Preparation:** In a small bowl, combine all sauce ingredients and stir till fully combined. If the sugar does not dissolve, place in the microwave for 30 seconds.
2. **Chop Vegetables:** Remove seeds from bell peppers and slice into strips. Next, slice the zucchini in half lengthwise, and then in half again. Chop into quarters.
3. **Stir-Fry Tofu:** Heat a pan over medium-high heat. Add 1 tablespoon oil, then stir-fry tofu until golden. Transfer tofu to a plate. Feel free to substitute tofu with any protein of your choice.
4. **Cook Vegetables:** Add 2 more tablespoons of oil to the pan. Stir-fry broccoli and green onions for about 3-5 minutes or until bright green, then add garlic, bell peppers, and zucchini. Pour in half of the sauce and cook until vegetables are tender.
5. **Finish Dish:** Return tofu to the pan, add the rest of the sauce, and cook until the sauce thickens.
6. **Serve:** Plate the stir-fry and enjoy!

Black Bean and Corn Quinoa Bowl

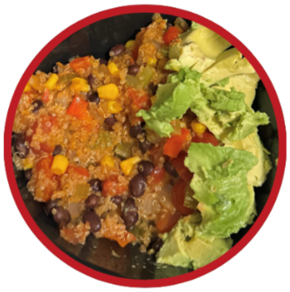

PREP TIME: 14 MIN

COOKING TIME: 24 MIN

SERVES: 4

Adapted from UC Davis Teaching Kitchen

Ingredients

- 2 tbsp oil
- 1 medium onion, chopped
- 1 medium bell pepper, chopped
- 1 celery rib, chopped
- 1 tsp salt
- 3 tsp smoked paprika
- 2 tsp garlic powder
- ¼ tsp pepper
- 2 cups vegetable stock
- 1 can corn, drained
- 1 can diced tomatoes, undrained
- 1 cup quinoa
- 1 can black beans, drained

Directions

1. **Chop Vegetables:** Finely chop the bell pepper, celery stick, and onion.
2. **Saute Vegetables:** Heat the oil in a pot, over medium heat. Add the chopped veggies, salt, smoked paprika, garlic powder, pepper and saute until the onion is translucent.
3. **Cook Quinoa Mixture:** Add the vegetable broth, quinoa, drained can of corn, and undrained can of diced tomatoes to the pot. Place the cover on the pot and let it simmer for 12-13 minutes or until the broth has been absorbed and the quinoa is cooked.
4. **Add Black Beans:** Once the quinoa is cooked, add the drained black beans. Continue with a low simmer to blend flavors.
5. **Serve: Adding toppings of your choice!** Enjoy!! * Recipe Suggestion: Cilantro or avocado*

On-Campus Food Resources

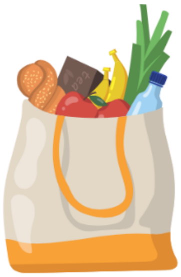

The Pantry EST. 2010

Get FREE Food on Campus!

Bring your student ID to the Pantry to get fresh items! The Pantry is now located next to the outdoor ATMs, across the road from Young Hall.

Need FREE Money for Groceries?

Scan the QR code to see if you qualify for up to **$291 a month** for groceries. For more info, visit Aggie Compass in the MU next to the Market

Aggiecompass.ucdavis.edu/calfresh
Aggiecompass.ucdavis.edu/aggiefresh

CalFresh

Table S1. Comparison of cooking self-efficacy pre- and post-measure.

Questions	Meal Kit Group (n=32)		*P*-Value	Recipe Group (n=4)		*P*-Value
	Pre-	Post-		Pre-	Post-	
How confident are you in being able to cook a nutritious meal?						
Not at all confident, n (%)	0 (0)	0 (0)	0.25	0 (0)	0 (0)	0.81
Slightly confident, n (%)	3 (9)	2 (6)		0 (0)	1 (25)	
Somewhat confident, n (%)	6 (19)	3 (9)		0 (0)	0 (0)	
Fairly confident, n (%)	14 (44)	17 (53)		2 (50)	0 (0)	
Completely confident, n (%)	9 (28)	10 (31)		2 (50)	3 (75)	
How confident are you in being able to cook a meal in a short period of time?						
Not at all confident, n (%)	0 (0)	0 (0)	0.31	0 (0)	0 (0)	0.50
Slightly confident, n (%)	6 (19)	3 (9)		0 (0)	0 (0)	
Somewhat confident, n (%)	10 (31)	8 (25)		1 (25)	3 (75)	
Fairly confident, n (%)	10 (31)	16 (50)		2 (50)	0 (0)	
Completely confident, n (%)	6 (19)	5 (16)		1 (25)	1 (25)	
How confident are you in being able to cook a nutritious meal on a budget?						
Not at all confident, n (%)	2 (6)	1 (3)	0.01*	0 (0)	0 (0)	0.18
Slightly confident, n (%)	3 (9)	2 (6)		0 (0)	0 (0)	
Somewhat confident, n (%)	18 (56)	9 (28)		0 (0)	1 (25)	
Fairly confident, n (%)	7 (22)	16 (50)		3 (75)	3 (75)	
Completely confident, n (%)	2 (6)	4 (13)		1 (25)	0 (0)	
How confident are you in being able to follow a recipe?						
Not at all confident, n (%)	1 (3)	0 (0)	0.01*	0 (0)	0 (0)	0.72
Slightly confident, n (%)	0 (0)	0 (0)		0 (0)	0 (0)	
Somewhat confident, n (%)	2 (6)	0 (0)		0 (0)	1 (25)	
Fairly confident, n (%)	12 (38)	10 (31)		2 (50)	1 (25)	
Completely confident, n (%)	17 (53)	22 (69)		2 (50)	2 (50)	
Summative Score (max = 20 points)						
	14.9 (± 2.8)	16.1 (± 2.4)	0.01*	17.2 (± 2.2)	15.8 (± 2.4)	0.53

*$p<0.05$

**Table S2.** Feedback for High-Protein Avocado Toast (recipe #1).		
	**Meal-Kit Group (n=41)**	**Recipe Group (n=12)**
Did you cook the recipe from the meal kit (or recipe card)?		
Yes, n (%)	38 (93)	11 (92)
No, n (%)	3 (7)	1 (8)
When using the meal kit (or recipe card), did you follow the provided recipe as intended?		
Yes, n (%)	39 (95)	9 (75)
No, n (%)	2 (5)	3 (25)
Approximately, how much of the food from the meal kit recipe (or recipe) did you eat?		
25%, n (%)	2 (5)	3 (25)
50%, n (%)	7 (17)	0 (0)
75%, n (%)	7 (17)	1 (8)
100%, n (%)	25 (61)	8 (67)
Approximately, how many meals did you find the recipe made?		
1 meal, n (%)	20 (49)	4 (33)
2 meals, n (%)	17 (41)	7 (59)
3 meals, n (%)	2 (5)	0 (0)
4 meals, n (%)	1(2)	0 (0)
5 meals, n (%)	0 (0)	0 (0)
Other, n (%)	1 (2)	1 (8)
Did you have any leftovers from the recipe?		
Yes, n (%)	17 (41)	5 (42)
No, n (%)	24 (59)	7 (58)
Were there specific ingredients within the recipe you did not consume?		
Yes, n (%)	6 (15)	4 (33)
No, n (%)	35 (85)	8 (67)
Did you enjoy the recipe?		
Yes, n (%)	34 (83)	7 (58)
No, n (%)	5 (12)	3 (25)
Not sure, n (%)	2 (5)	2 (17)
Did you have all of the cooking equipment needed to prepare this recipe?		
Yes, n (%)	40 (98)	12 (100)
No, n (%)	1 (2)	0 (0)
How easy was it to make this recipe?		
Very easy, n (%)	35(85)	6 (50)
Somewhat easy, n (%)	5 (12)	3 (25)
Somewhat difficult, n (%)	1 (2)	1 (8)
Very difficult, n (%)	0 (0)	0 (0)
Unreported, n (%)	0 (0)	2 (17)
How likely are you to cook this recipe again on your own?		
Very likely, n (%)	17 (41)	4 (33)
Somewhat likely, n (%)	14 (34)	3 (25)
Somewhat unlikely, n (%)	4 (10)	1 (8)

Very unlikely, n (%)	6 (15)	2 (17)
Unreported, n (%)	0 (0)	2 (17)
Would you recommend this recipe to a friend or family member?		
Very likely, n (%)	21 (51)	4 (33)
Somewhat likely, n (%)	14 (34)	4 (33)
Somewhat unlikely, n (%)	3 (7)	2 (17)
Very unlikely, n (%)	3 (7)	0 (0)
Unreported, n (%)	0 (0)	2 (17)

**Table S3.** Feedback for the Korean Vegetable Tofu Stir Fry (recipe #2).		
	**Meal-Kit Group (n=42)**	**Recipe Group (n=7)**
Did you cook the recipe from the meal kit (or recipe card)?		
Yes, n (%)	42 (100)	7 (100)
No, n (%)	0 (0)	0 (0)
When using the meal kit (or recipe card), did you follow the provided recipe as intended?		
Yes, n (%)	42 (100)	7 (100)
No, n (%)	0 (0)	0 (0)
Approximately, how much of the food from the meal kit recipe (or recipe) did you eat?		
25%, n (%)	3 (7)	1 (14)
50%, n (%)	5 (12)	1 (14)
75%, n (%)	7 (17)	3 (43)
100%, n (%)	26 (62)	2 (29)
Unreported, n (%)	1 (2)	0 (0)
Approximately, how many meals did you find the recipe made?		
1 meal, n (%)	10 (24)	1 (14)
2 meals, n (%)	13 (31)	5 (71)
3 meals, n (%)	15 (36)	1 (14)
4 meals, n (%)	3 (7)	0 (0)
5 meals, n (%)	0 (0)	0 (0)
Other, n (%)	1 (2)	0 (0)
Did you have any leftovers from the recipe?		
Yes, n (%)	25 (60)	6 (86)
No, n (%)	17 (40)	1 (14)
Were there specific ingredients within the recipe you did not consume?		
Yes, n (%)	11 (26)	2 (29)
No, n (%)	31 (74)	5 (71)
Did you enjoy the recipe?		
Yes, n (%)	33 (78)	3 (43)
No, n (%)	2 (5)	1 (14)
Not sure, n (%)	5 (12)	3 (43)
Unreported, n (%)	2 (5)	0 (0)

Did you have all of the cooking equipment needed to prepare this recipe?		
Yes, n (%)	40 (95)	7 (100)
No, n (%)	0 (0)	0 (0)
Unreported, n (%)	2 (5)	0 (0)
How easy was it to make this recipe?		
Very easy, n (%)	16 (38)	3 (43)
Somewhat easy, n (%)	20 (48)	3 (43)
Somewhat difficult, n (%)	4 (9)	1 (14)
Very difficult, n (%)	0 (0)	0 (0)
Unreported, n (%)	2 (5)	0 (0)
How likely are you to cook this recipe again on your own?		
Very likely, n (%)	10 (24)	0 (0)
Somewhat likely, n (%)	18 (43)	5 (71)
Somewhat unlikely, n (%)	9 (21)	1 (14)
Very unlikely, n (%)	3 (7)	1 (14)
Unreported, n (%)	2 (5)	0 (0)
Would you recommend this recipe to a friend or family member?		
Very likely, n (%)	15 (35)	2 (29)
Somewhat likely, n (%)	18 (43)	5 (71)
Somewhat unlikely, n (%)	5 (12)	0 (0)
Very unlikely, n (%)	2 (5)	0 (0)
Unreported, n (%)	2 (5)	0 (0)

**Table S4.** Feedback for the Mexican-Inspired Quinoa Bowl (recipe #3).		
	**Meal-Kit Group (n=38)**	**Recipe Group (n=6)**
Did you cook the recipe from the meal kit (or recipe card)?		
Yes, n (%)	38 (100)	6 (100)
No, n (%)	0 (0)	0 (0)
When using the meal kit (or recipe card), did you follow the provided recipe as intended?		
Yes, n (%)	35 (92)	6 (100)
No, n (%)	3 (8)	0 (0)
Approximately, how much of the food from the meal kit recipe (or recipe) did you eat?		
25%, n (%)	10 (26)	1 (17)
50%, n (%)	8 (21)	2 (33)
75%, n (%)	9 (24)	1 (17)
100%, n (%)	11 (29)	2 (33)
Approximately, how many meals did you find the recipe made?		
1 meal, n (%)	3 (8)	1 (17)
2 meals, n (%)	4 (11)	4 (67)
3 meals, n (%)	7 (18)	1 (17)
4 meals, n (%)	11 (29)	0 (0)
5 meals, n (%)	9 (24)	0 (0)
Other, n (%)	4 (11)	0 (0)
Did you have any leftovers from the recipe?		
Yes, n (%)	29 (76)	5 (83)
No, n (%)	9 (24)	1 (17)
Were there specific ingredients within the recipe you did not consume?		
Yes, n (%)	5 (13)	2 (33)
No, n (%)	33 (87)	4 (67)
Did you enjoy the recipe?		
Yes, n (%)	31 (82)	4 (67)
No, n (%)	2 (5)	1 (17)
Not sure, n (%)	5 (13)	1 (17)
Did you have all of the cooking equipment needed to prepare this recipe?		
Yes, n (%)	35 (92)	6 (100)
No, n (%)	3 (8)	0 (0)
How easy was it to make this recipe?		
Very easy, n (%)	20 (53)	3 (50)
Somewhat easy, n (%)	15 (39)	3 (50)
Somewhat difficult, n (%)	3 (8)	0 (0)
Very difficult, n (%)	0 (0)	0 (0)
How likely are you to cook this recipe again on your own?		
Very likely, n (%)	12 (32)	2 (33)
Somewhat likely, n (%)	11 (29)	2 (33)

Somewhat unlikely, n (%)	9 (24)	2 (33)
Very unlikely, n (%)	6 (16)	0 (0)
Would you recommend this recipe to a friend or family member?		
Very likely, n (%)	16 (42)	3 (50)
Somewhat likely, n (%)	9 (24)	3 (50)
Somewhat unlikely, n (%)	10 (26)	0 (0)
Very unlikely, n (%)	3 (8)	0 (0)

Table S5: Qualitative feedback from participants.

**Themes**	**Sub Themes**	**Exemplar Quotes**
Convenience and Ease of Use	Minimal preparation or difficulty	"It didn't require much preparation or cooking. I learned that I can make a delicious and nutritious meal under budget and with limited kitchen supplies. It was also a fast process."- Meal Kit #1 Participant<br><br>"I would recommend this meal because it was incredibly flavorful and had nice veggies. It's easy to make, has nice flavors, and easy to clean. If I had a friend struggling on making simple meal ideas that doesn't involve laborious prep work or constant stirring, I would direct them to this recipe."- Meal Kit #3 Participant
	Fast/convenient/quick/easy methodology and instruction	"I really liked how most ingredients were pre-measured and made it fun and easier to follow the recipe."- Meal Kit #1 Participant<br><br>"I liked how easy it was to prepare, and that the ingredients were clearly marked and separated."- Meal Kit #3 Participant<br><br>"It felt healthy, filling, and had a lot of flavors so I didn't feel like I was missing out…It is also easy to incorporate into my schedule since it just takes time to assemble and toast."- Meal Kit #1 Participant<br><br>"My family typically enjoys rice as our main diet but we do indulge in sandwiches for mornings and quick bites--since this is easy to assemble and has decent nutritional value, I would be more than happy to recommend this to my family so we can all enjoy it together." - Meal Kit #1 Participant<br><br>"I liked how the recipe used canned goods. It seems like canned corn and canned black beans are commonly donated to free food pantries, so they will be easy to find for free too."- Meal Kit #3 Participant<br><br>"Since it was so easy, satiating, and nutritious to make, I would love to make this meal again. It also made a lot of portions, so I was able to pack half of the recipe into my

		fridge to eat the day after I made the meal in its entirety."- Meal Kit #3 Participant
Sensory Appeal and Nutritional Value	Positive sensory appeal (texture, taste, smell, appearance)	"I wasn't a big fan of the raw tofu at first but then I began to really like it..."- Meal Kit #1 Participant<br><br>"I liked that I was able to try new things. It was my first time trying nutritional yeast and I really liked the avocado and crumbled tofu combination."- Meal Kit #1 Participant<br><br>"The diversity in taste, texture, and variety in the sauce itself was really enjoyable. I think it challenges the stereotype of vegan/vegetarian food being bland or just leafy greens. The crunchiness of the bell pepper and zucchini complemented the spring onions and softness of the tofu."- Meal Kit #2 Participant
	Negative sensory appeal (texture, taste, smell, appearance)	"Personally I just didn't like the taste of the sauce. But it made me realize that I did like fried tofu."- Meal Kit #2 Participant<br><br>"I least liked the seasoning of the toast because I somewhat dislike nutritional yeast in my food."- Meal Kit #1 Participant<br><br>"I did not really like the texture of the tofu but it was still very delicious so I got over it (kind of)."- Meal Kit #1 Participant<br><br>"I think the ginger taste might be off putting to people who aren't used to having such strong flavors alongside the gochujang, especially because it was a lot of ginger to put in with the vegetables when it has such as strong flavor profile."- Meal Kit #2 Participant<br><br>"I liked least that the recipe called for canned goods. I usually stay away from canned things."- Meal Kit #3 Participant<br><br>"It is unlikely that I buy all these ingredients because these are not things that my family usually eat."- Meal Kit #3 Participant

	Nutrient-dense or healthy meals	"It motivated me to have a high protein breakfast."- Meal Kit #1 Participant<br><br>"This meal is a great way to meet my protein goals without consuming a ton of cholesterol through eggs, etc."- Meal Kit #1 Participant<br><br>"I plan to make this recipe for my family as it is very healthy yet there is lots of flavor. I believe they would thoroughly enjoy it."- Meal Kit #2 Participant<br><br>"Since it was so easy, satiating, and nutritious to make, I would love to make this meal again. It also made a lot of portions, so I was able to pack half of the recipe into my fridge to eat the day after I made the meal in its entirety."- Meal Kit #3 Participant<br><br>"My dad's doctor recommended him to eat quinoa, but he was never too fond of the taste. I believe this recipe will help him eat healthier and enjoy a tasteful meal."- Meal Kit #3 Participant
Ingredient Familiarity and Accessibility	Familiarity with ingredients	"I am already a fan of avocado toast, nutritional yeast, and tofu, so I really enjoyed this high-protein twist on the popular dish."- Meal Kit #1 Participant<br><br>"It was really good. I usually have plain avocado toast. It was really good to have an option with higher protein."- Meal Kit #1 Participant<br><br>" It was delicious and decently easy to make with ingredients I typically enjoy already."- Meal Kit #2 Participant<br><br>"I really enjoy quinoa salads so it was cool to try a new quinoa recipe. It was very taste and I look forward to implementing this recipe into my quinoa recipes."- Meal Kit #3 Participant
	Hard-to-acquire ingredients or dislike of specific ingredients	"It is quick, but expensive to collect the ingredients the first time."- Meal Kit #1 Participant<br><br>"Not a big fan of stir fry in general and I hate zucchini."- Meal Kit #2 Participant

		"It was not difficult to cook this recipe but I might have difficulty getting all the ingredients for the sauce."- Meal Kit #2 Participant<br><br>"I would cook it again. I would just need to get all the ingredients together, which would be the more difficult part."- Meal Kit #2 Participant<br><br>"Unlikely to have all ingredients necessary and have never used gochujang in other recipes."- Meal Kit #2 Participant<br><br>"Most of my friends and family consume a lot of cultural foods and are unlikely to try any new foods outside of what they're familiar with."-Meal Kit #3 Participant
	Introduction of new ingredients and recipes with a positive outlook	"I actually have been having a hard time eating ANY food and have been losing weight. I was very surprised when I liked the toast and it made me happy because I didn't have to force it down like I have been doing. I think the flavor profile was very different and exciting because I feel like maybe I just need to venture out more with food."- Meal Kit #1 Participant<br><br>"I never had tofu raw and just added on with avocado as a sort of version of avocado toast for breakfast so this was an interesting recipe to try! I think I would do it again but perhaps if I had prepped prior, I might try baking the tofu in the oven or stir frying it with seasonings to enhance it. As the current recipe, it's easy to make and quick to do so I think I'd make it again!"- Meal Kit #1 Participant<br><br>"This was my first time cooking quinoa. It is not something I usually eat because I typically cook white rice. But it was still delicious."- Meal Kit #3 Participant
Cooking Skills and Equipment	Lack of cooking knowledge	"I don't have much cooking experience so it took me some time to read and understand the instructions but overall, it was not difficult."- Meal Kit #2 Participant<br><br>"I do not know how to cut an onion, but besides that everything was pretty self-explanatory."- Meal Kit #3 Participant

		"Instructions were unclear on part about cooking quinoa - when simmering do you need to bring it to a boil first? or just simmer the whole time?"- Meal Kit #3 Participant<br><br>"This was my first time cooking quinoa so it was first time experience. Also, poor pot estimation made the cooking process take longer than needed. "- Meal Kit #3 Participant<br><br>"I do not know how to mince and I suck at cutting vegetables so that took up most of my time."- Meal Kit #2 Participant<br><br>"Although I followed the recipe, it's hard to tell if the broccoli is cooked well before I taste it."- Meal Kit #2 Participant
	Missing or insufficient cooking equipment	"It was simple, it just took me a while to make it, but that's probably because I have smaller sized stove tops and cut the tofu into too many pieces, resulting in me cooking the tofu in sections instead of all at once. I also had to go grocery shopping for the sauce ingredients because I didn't have any and now I don't know what to do with the red vinegar, but I'm glad I have it."- Meal Kit #2 Participant<br><br>"Pan was too small."- Meal Kit #2 Participant
	Increase confidence in cooking skills	"I liked the sauce, it made me feel confident about making other sauces."- Meal Kit #2 Participant<br><br>"After making it once I feel like I can be able to replicate it, or even do a better job at following the recipe. The ingredients needed are not difficult to find and the serving sizes are just fine."- Meal Kit #2 Participant<br><br>"I liked cooking it because it actually motivated me to try new things and practice skills I do not have."- Meal Kit #2 Participant
Recipe Modifications	Made modifications or planned future changes	"I actually had a bunch of leftover meat and the stir-fry was just what I needed to have something to eat with meat. I appreciate that the recipe card recommended using meat as a substitute for the tofu if I wanted to."- Meal Kit #2 Participant

		"I enjoyed the overall taste and how it reminds me of something my dad would make at home but if I made this again I wouldn’t cook the bell peppers or tomatoes. I personally enjoy a variety of textures in my meal, but sometimes I won’t mind a single textured meal, it depends on how I’m feeling."- Meal Kit #3 Participant<br><br>"As I mentioned, the meal is very meal prep worthy and customizable. In my left-over meals, I roasted some chickpeas for crunchy toppings and that went really well with it. I added avocado and cilantro. With the amount of meals I got out of it, I’d say it’s worth to make again!"- Meal Kit #3 Participant
	Adaptability of recipes to meet dietary or personal preferences	"I have vegetarian friends who are looking for simple and delicious recipes. This is definitely a recipe I would recommend to them as it is tasty and easy to make."- Meal Kit #2 Participant<br><br>"If my family or friends who are vegan asked for a recipe that involves giving them a complete protein diet, then I would recommend them this recipe. But if they don't know what nutritional yeast is or taste like, I would warn them about the taste."- Meal Kit #1 Participant
General Positive or Negative Perception	General positive feedback (confidence increase, good experience)	"I like the amount of food the recipe yields and the ingredients are cheap so I'll make it again"- Meal Kit #3 Participant<br><br>"I like the process of cooking it, it is rather fun."- Meal Kit #3 Participant<br><br>"The recipe called for foods that I usually have other than quinoa, but I also have some of it right now, so if I feel like it and have all the ingredients or maybe just some, I might make it or something similar based on the recipe."- Meal Kit #3 Participant
	General negative feedback (time-consuming, not easy)	"There are foods I like more that use similar ingredients, don't like vegetable dishes that much."- Meal Kit #1 Participant<br><br>"There are foods I like more that use similar ingredients." - Meal Kit #2 Participant

	Sharing meals with others	"I would definitely share this recipe with my mom she loves finding new recipes full of vegetables."- Meal Kit #2 Participant<br><br>"I sent the recipe to my boyfriend as I was making it."- Meal Kit #2 Participant<br><br>"I have friends that are vegetarian/vegan and I think this would be a great breakfast item for them."- Meal Kit #1 Participant<br><br>"I already told my sister and boyfriend about this because I thought the combination was interesting."- Meal Kit #1 Participant<br><br>"I thought this tasted good and I have a lot of vegetarian friends who are looking to increase their protein intake."- Meal Kit #1 Participant<br><br>"My mom also enjoys quinoa so I shared this recipe with her and hopefully she will try it out and let me know if she also enjoys it."-Meal Kit #3 Participant<br><br>"My brother came to visit and tried some and he really liked it! But before he tried it, I definitely would have shared it because of how it tasted and easy to make it was."- Meal Kit #3 Participant
	Social Value	"I was thinking that you guys should make video descriptions on how to make the recipes. Since I am a novice cook, sometimes I was unsure how things were supposed to look. I am sure there are others like me or even worse."- Meal Kit #2 Participant<br><br>"I wish whoever made this up had an Instagram page with recipes, even if it is a UCD page. I have been buying all of my favorite foods and practically force feeding myself. I think sharing stuff like this could help people who struggle with food and even those that don't."- Meal Kit #1 Participant
